# Pre-Liver Transplant: Tips Versus Distal Splenorenal Shunt

**DOI:** 10.1155/1997/41536

**Published:** 1997

**Authors:** Thomas W. Faust, Michael F. Sorrell

**Affiliations:** University of Nebraska Medical Center Liver Transplant Program 600 S. 42nd Street Omaha Nebraska 68198-3280 USA

## Abstract

Recurrent variceal bleeding in liver transplant candidates with end-stage liver disease can complicate or even prohibit a subsequent transplant procedure (OLT). Endoscopic sclero-therapy and medical therapy are considered as first-line management with surgical shunts reserved for refractory situations. Surgical shunts can be associated with a high mortality in this population and may complicate subsequent OLT. The transjugular intrahepatic portosystemic shunt (TIPS) has been recommended in these patients as a bridge to OLT. This is a new modality that has not been compared with previously established therapies such as the distal splenorenal shunt (DSRS). In this study we report our experience with 35 liver transplant recipients who had a previous TIPS (18 patients) or DSRS (17 patients) for variceal bleeding. The TIPS group had a significantly larger proportion of critically ill and Child-Pugh C patients. Mean operating time was more prolonged in the DSRS group (*P*=0.014) but transfusion requirements were similar. Intraoperative portal vein blood flow measurements averaged 2132±725 ml/min in the TIPS group compared with 1120±351ml/min in the DSRS group (*P*<0.001). Arterial flows were similar. Mean ICU and hospital stays were similar. There were 3 hospital mortalities in the DSRS group and none in the TIPS group (*P*=0.1). We conclude that TIPS is a valuable tool in the management of recurrent variceal bleeding prior to liver transplantation. Intra0Perative hemodynamic measurements suggest a theoretical advantage with TIPS. In a group of patients with advanced liver disease we report an outcome that is similar to patients treated with DSRS prior to liver transplantation. The role of TIPS in the treatment of nontransplant candidates remains to be clarified.


[5] Scheele, J., Stangl, R., Altendorf-Hofmann, A. and Paul, M. (1995). Resection of colorectal liver metastases. *World Journal of Surgery*, **19**, 59–71.
[6] Doci, R., Gennari, L., Bignami, P., Montalto, F., Morabito, A. and Bozzetti, F. (1991). One hundred patients with hepatic metastases from colorectal cancer treated by resection: analysis of prognostic determinants. *British Journal of Surgery*, **78**, 797–801.
[7] Yanaga, K., Kanematsu, T. and Takenaka, K. *et al.* (1988). Hepatic resection for hepatocellular carcinoma in elderly patients. *American Journal of Surgery*, **155**, 238–241.
[8] Fortner, J.G. and Lincer, R.M. (1990). Hepatic resection in the elderly. *Annals of Surgery*, **211**, 141–145.
[9] Nagasue, N., Chang, Y.C. and Takemoto, Y. *et al.* (1993). Liver resection in the aged (seventy years or older) with hepatocellular carcinoma. *Surgery*, **113**, 148–154.

O. J. Garden, MD, FRCS
Senior Lecturer in Surgery
Director of Organ Transplantation
University Department of Surgery
Royal Infirmary, Lauriston Place
Edinburgh
United Kingdom

### Pre-Liver Transplant: Tips Versus Distal Splenorenal Shunt

### ABSTRACT

*Abouljoud, M.S., Levy, M.F., Rees, C.R., Diamond, N.G., Lee, S.P., Mulligan, D.C., Goldstein, R.M., Husberg, B., Gonwa, T.A. and Klintmalm, G.B. (1995)A comparison of treatment with transjugular intrahepatic portosystemic shunt or distal splenorenal shunt in the management of variceal bleeding prior to liver transplantation. Transplantation, 59: 226–229.*

**Recurrent variceal bleeding in liver transplant candidates with end-stage liver disease can complicate or even prohibit a subsequent transplant procedure (OLT). Endoscopic sclero-therapy and medical therapy are considered as first-line management with surgical shunts reserved for refractory situations. Surgical shunts can be associated with a high mortality in this population and may complicate subsequent OLT. The transjugular intrahepatic portosystemic shunt (TIPS) has been recommended in these patients as a bridge to OLT. This is a new modality that has not been compared with previously established therapies such as the distal splenorenal shunt (DSRS). In this study we report our experience with 35 liver transplant recipients who had a previous TIPS (18 patients) or DSRS (17 patients) for variceal bleeding. The TIPS group had a significantly larger proportion of critically ill and Child-Pugh C patients. Mean operating time was more prolonged in the DSRS group ($P$=0.014) but transfusion requirements were similar. Intraoperative portal vein blood flow measurements averaged 2132±725 ml/min in the TIPS group compared with 1120±351ml/min in the DSRS group ($P$<0.001). Arterial flows were similar. Mean ICU and hospital stays were similar. There were 3 hospital mortalities in the DSRS group and none in the TIPS group ($P$=0.1). We conclude that TIPS is a valuable tool in the management of recurrent variceal bleeding prior to liver transplantation. Intraoperative hemodynamic measurements suggest a theoretical advantage with TIPS. In a group of patients with advanced liver disease we report an outcome that is similar to patients treated with DSRS prior to liver transplantation. The role of TIPS in the treatment of nontransplant candidates remains to be clarified.**

*Keywords*: TIPS, distal spenorenal shunt, liver transplant

### PAPER DISCUSSION

The paper by Abouljoud, *et al.* describes their experience with the use of transjugular intrahepatic portosystemic shunt (TIPS) in a busy transplant service and compares distal splenorenal shunts (DSRS) to TIPS relative to post-transplant outcome. Variceal bleeding in patients with portal hypertension is a frequent finding in patients with end stage liver disease [1–4]. It is critically important to assess risk factors for bleeding and to use preventive strategies in the evaluation and management of patients with known varices in the patient awaiting transplantation since variceal bleeding may exclude or complicate liver transplantation. Therapeutic options for treatment of bleeding esophagogastric varices and prevention of rebleeding should be structured to optimize patient outcome and assure successful transplantation.

Risk factors for predicting hemorrhage from varices have been well defined. Variceal size, intravariceal pressure, red color signs and the Child Pugh classification of liver disease are important variables in the evaluation of patients at risk for hemorrhage [5]. Lebrec *et al.* have shown that a portal or hepatic venous pressure gradient of at least 12 mm Hg is required for the developement of hemorrhage from esophageal varices, but that gradations in pressure above 12 mm Hg are not associated with a proportional increase in bleeding risk [6].

Prevention of variceal bleeding is important because mortality dramatically increases after bleeding occurs. Non-selective beta blockers may be used to prevent the first variceal hemorrhage in patients with end stage liver disease; patients with decompensated liver disease and medium to large varices are most likely to benefit [7,8]. Prevention of initial variceal hemorrhage through shunt surgery is not beneficial. Prophylatic sclerotherapy of varices is also not an effective preventive strategy [8,9]. Patients with end stage liver disease who bleed from esophageal varices warrant aggressive treatment to control the initial bleed and to prevent rebleeding. Acute bleeding may be controlled endoscopically with either sclerotherapy or band ligation [2–4,10]. Endoscopic ligation of varices controls acute bleeding as effectively as sclerotherapy, but with fewer complications, reduced rebleeding rates, and possibly improved survival [10]. Well-known complications associated with endoscopic treatment include esophageal ulceration or perforation, cardiopulmonary sequelae, and infections, all of which may preclude transplantation or increase operative risk [10–12]. Additionally, vasoactive agents may be used for the acute treatment of bleeding varices [13]. Vasopressin with nitroglycerin, glypressin, somatostatin, and octreotide may effectively control variceal hemorrhage, but only glypressin has been shown to significantly improve survival. In the patient who has recovered from a variceal bleed, the addition of non-selective beta blockers to endoscopic therapy reduces rebleeding rates when compared to either therapy alone but mortality rates may not be significantly improved [14,15].

What options are available for patients who continue to experience variceal bleeding despite sclerotherapy, variceal ligation and pharmacologic intervention? Both DSRS and TIPS control variceal hemorrhage and prevent rebleeding in over 90% of cases. As with all therapies, risks and benefits must be carefully considered. Rebleeding rates and patient survival are not significantly different between selective and non-selective shunts but the incidence of hepatic encephalopathy may be less in patients receiving DSRS [16–18].

TIPS controls acute variceal bleeding and prevents rebleeding in patients refractory to standard medical and endoscopic therapy. [19–22] TIPS has been associated with a variety of complications [20–22]. Fifteen to 66% of patients may develop shunt stenosis or occlusion and recurrent variceal bleeding within 1 year following TIPS placement. Seven to 30% of patients may experience new or worsening encephalopathy. In addition, improperly placed TIPS may also increase the difficulty of the transplant operation [23]. Potential complications resulting from TIPS should be carefully considered, keeping in mind the effect that complications may have on candidacy for liver transplant. TIPS is a short-term solution for the prevention of recurrent hemorrhage in patients with end stage liver disease who are candidates for transplantation.

What factors are important to consider in management of the potential transplant candidate with variceal hemorrhage? Several factors including the clinical status of the patient, the etiology and severity of liver disease, and candidacy for liver transplantation should be assessed before shunting or placement of TIPS. The etiology of liver disease influences survival of the patient with variceal bleeding. Patients with cholestatic liver disease tolerate variceal bleeding better than those with parenchymal liver disease [24, 25]. Severity of liver disease is important in determining whether to use DSRS or TIPS. Survival in patients with compensated liver disease receiving DSRS for variceal bleeding is superior to that of patients with decompensated liver disease [26]. Survival in patients who are treated with DSRS in the setting of mild to moderate liver disease is comparable to that of patients receiving allografts for a similar degree of liver failure, but patients with decompensated liver disease benefit more from transplantation [27, 28]. Surgical shunting should be reserved for patients with compensated liver disease and hemorrhage refractory to nonsurgical methods. TIPS followed by timely transplantation should be considered for patients with refractory variceal bleeding in the setting of decompensated liver disease [19, 20].

While transplantation is an effective therapy for end-stage liver disease; management of variceal hemorrhage needs to be carefully individualized to optimize patient outcome after transplantation. Abouljoud *et al.* describe their data comparing TIPS and DSRS in regards to safety, efficacy, long-term complications and influence on subsequent liver transplantation. Outcome parameters included operating time, number of transfusions, intraoperative portal venous and hepatic arterial flow measurements; length of stay and operative mortality was also assessed. Mean operating time was significantly longer in the DSRS group but there was no significant difference in transfusion requirements or cold ischemia times between the groups. Portal venous flow was significantly reduced in patients receiving DSRS compared to patients receiving TIPS but there was no significant difference in hepatic arterial flow between the groups. Analysis of the data demonstrated that patients undergoing TIPS has a similar post-operative course in terms of mortality, ICU and hospital stay compared to those patients who had DSRS even though they had significantly worse liver disease. Although not statistically significant, it is worth noting that 3 deaths occurred in the DSRS population and no deaths occurred in the TIPS group. The authors conclude that TIPS is a valuable tool in the management of patients with severe end stage liver disease awaiting transplantation who present with variceal hemorrhage.

A number of questions regarding methodology are raised in reviewing the paper by Abouljoud and colleagues that if clarified would be helpful in further defining the role of DSRS vs TIPS as adjunctive therapy in the potential transplant patient. The definition of refractory variceal hemorrhage was not provided. The authors state that follow-up ranged from 1–96 months. It would be helpful to know the duration of follow-up prior to and after liver transplantation. Recent studies suggest that 30 day post-TIPS survival in the non-transplant candidate with Child-Pugh class C liver disease and variceal bleeding is considerably worse when compared to that in patients with milder liver disease [29,30]. TIPS followed by timely transplantation in this patient population reduces mortality rates; this may account for the promising results in patients with Child-Pugh class C liver disease receiving TIPS.

What practical conclusions can we draw from Abjoulboud's experience? Their data would suggest that the use of TIPS can safely serve as a bridge to transplantation and is particularly useful in the patient with advanced liver disease. Although the numbers were too small to draw firm statistical conclusions, there were three hospital mortalities in the DSRS group and none in the TIPS group even though there was a larger proportion of critically ill and Child-Pugh class C patients in the TIPS group. However, the role of DSRS in the treatment of variceal hemorrhage should not be minimized. In the patient with relatively well preserved synthetic function and recurrent variceal hemorrhage, DSRS is a time-tested, proven and effective treatment. The application of a less-invasive treatment modality in the hemorrhaging liver patient adds another alternative to our arsenal of therapeutic options. As is the case in all studies of new treatment modalities, the study suffers from small numbers in each group. While the results of Abouljoud and colleagues are encouraging a larger, prospective, randomized, controlled trial comparing endpoints of efficacy and safety of DSRS and TIPS would be helpful in defining standards of practice in the patient awaiting transplantation.

### *References*

[1] Navarro, V.J. and Garcia-Tsao, G. (1995). Varicealhemorrhage. *Critical Care Clinics*, **11**, 391–414.

[2] Terblanche, J., Burroughs, A.K. and Hobbs, K.E. (1989). Controversies in the management of bleeding esophageal varices. *New England Journal of Medicine*, **320**, 1393–1398, 1469–1475.

[3] Lebrec, D. (1992). Portal hypertension in complications of Chronic Liver Disease, edited by Rector, W. pp. 24–67, St. Louis: Mosby-Year Book, Inc.

[4] Reyes, J., Irvatsuki, S. (1992). Current management of portal hypertension with liver transplantation. *Advances in Surgery*, **25**, 189–208.

[5] The North Italian Endoscopic Club for the Study and Treatment of Esophageal Varices (1988). Prediction of the first variceal hemorrhage in patients with cirrhosis of the liver and esophageal varices: A prospective multi-center study. *New England Journal of Medicine*, **319**, 983–989.

[6] Lebrec, D., Fleury, O., Reu, B., Nahum, H. and Benhamou, J.P. (1980). Portal hypertension, size of esophageal varices and risk of gastrointestinal bleeding in alcoholic cirrhosis. *Gastroenterology*, **79**, 1139–1144.

[7] Poynard, T., Cales, P., Pasta, L., Ideo, G., Pascal, J.P., Pagliaro, L. and Lebrec, D. (1991). Beta-adrenergic-antagonist drugs in the prevention of gastrointestinal bleeding in patients with cirrhosis and esophageal varices: an analysis of data and prognostic factors in 589 patients from four randomized clinical trials. *New England Journal of Medicine*, **32**, 1532–1538.

[8] Pagliaro, L., D'Amico, G., Sorenson, T.I.A., Lebrec, D., Burroughs, A.K., Morabito, A., Tine, F., Politi, F. and Traina, M. (1992). Prevention of first bleeding in cirrhosis: a meta-analysis of randomized trials of nonsurgical treatment. *Annals of Internal Medicine*, **117**, 59–70.

[9] Van Ruiswyk, J. and Byrd, J.C. (1992). Efficacy of sclerotherapy for prevention of a first variceal hemorrhage. *Gastroenterology*, **102**, 587–597.

[10] Stiegmann, G.V., Go, J.A., Michaltez-Onody, P.A., Korula, J., Lieberman, D., Saeed, Z.A. and Reveille, M.R. (1992). Endoscopic scletotherapy as compared with endoscopic ligation for bleeding esophageal varices. *New England Journal of Medicine*, **326**, 1527–1532.

[11] Health and Public Policy Committee (1984). Endoscopic sclerotherapy for esophageal varices. *Annals of Internal Medicine*, **100**, 608–610.

[12] Gimson, A.E., Ramage, J.K., Panos, M.Z., Hayllar, K., Harrison, P.M., Williams, R. and Westaby, D. (1993). Randomized trial of variceal banding ligation versus injection sclerotherapy for bleeding oesophageal varices. *The Lancet*, **342**, 391–394.

[13] D'Amico, G., Luigi, P. and Bosch, J. (1995). The treatment of portal hypertension: A meta-analytic review. *Hepatology*, **22**, 332–354.

[14] Jensen, L.S. and Krarup, N. (1989). Propranolol in the prevention of rebleeding from esophageal varices during the course of endoscopic sclerotherapy. *Scandinavian Journal of Gastroenterology*, **24**, 339–345.

[15] Ink, O., Martin, T., Poynard, T., Reville, M., Anciaux, M.L., Lenoir, C., Marill, J.L., Lebadie, H., Masliah, C. and Perrin, D. (1992). Does elective sclerotherapy improve the efficacy of long term propranolol for prevention of recurrent bleeding in patients with severe cirrhosis? A prospective multi-center randomized trial. *Hepatology*, **16**, 912–919.

[16] Rikkers, L., Rudman, D. and Galambos, J. (1978). A randomized controlled trial of distal splenorenal shunts. *Annals of Surgery*, **188**, 271–282.

[17] Reichle, F., Fahmy, W. and Golsorkhi, M. (1979). Propsective comparative clinical trial with distal splenorenal and mesocaval shunts. *American Journal of Surgery*, **137**, 13–21.

[18] Millikan, W.,Warren, W., Henderson, J., Smith, R.B. III, Salam, A.A., Galambos, J.T., Kutner, M.H. and Keen, J.H. (1985). The Emory prospective randomized trial: Selective versus nonselective shunt to control variceal bleeding. Ten year follow-up. *Annals of Surgery*, **201**, 712–722.

[19] Ring, E., Lake, J., Roberts, J., Gordon, R., LaBerge, J., Read, A., Sterneck, E. and Ascher, N. (1992). Using transjugular intrahepatic portosystemic shunts to control variceal bleeding before liver transplantation. *Annals of Internal Medicine*, **116**, 304–306.
[20] Rossle, M., Haag, K., Ochs, A., Sellinger, M., Noldge, G., Perarneu, J., Berger, E., Blum, V., Gabelmann, A. and Hauenstein, K. (1994). The trasjugular intrahepatic portosystemic stent shunt procedure for variceal bleeding. *New England Journal of Medicine*, **330**, 165–171.
[21] LaBerge, J.M., Ring, E., Gordon, R., Lake, J., Doherty, M., Somberg, K., Roberts, J. and Ascher, N. (1993). Creation of transjugular intrahepatic portosystemic shunts with the wallstent endoprosthesis: Results in 100 patients. *Radiology*, **187**, 413–420.
[22] Shiman, M., Jeers, L., Hoofnagle, J. and Tralka, T. (1995). The role of transjugular intrahepatic shunt (TIPS) for treatment of portal hypertension and its complications. National Digestive Diseases Advisory Board. *Hepatology*, **22**, 1591–1597.
[23] Millis, J.M., Martin, P., Gomes, A., Shaked, A., Coliquhoun, S.D., Jurim, O., Goldstein, L. and Busuttil, R.W. (1995). Transjugular intrahepatic portosystemic shunts: Impact on liver transplantation. Liver Transplantation and Surgery, **1**, 229–233.
[24] Gores, G.J., Wiesner, R.H., Dickson, E.R., Zinmeister, A.R., Jorgensen, R.A. and Langworthy, A. (1989). Prospective Evaluation of esophageal varices in primary biliary cirrhosis. *Gastroenterology*, **96**, 1552–1559.
[25] Graham, D.Y. and Smith, J.L. (1981). The course of patients after variceal hemorrhage. *Gastroenterology*, **80**, 800–809.
[27] Wood, P., Shaw, B. and Rikkers, L. (1990). Liver transplantation for variceal hemorrhage. Surgical Clinics of North America, **70**, 449–461.
[28] Bismuth, H., Adam, R., Mathur, S. and Sherlock, D. (1990). Options for elective treatment of portal hypertension in cirrhotic patients in the transplantation era. *American Journal of Surgery*, **160**, 105–110.
[29] Coldwell, D.M., Ring, E.J., Rees, C.R., Zemel, G., Darcy, M.D., Haskal, Z.J., McKusick, M.A. and Greenfield, A.J. (1995). Multi-center investigation of the role of transjugular intrahepatic portosystemic shunt in the management of portal hypertension. *Radiology*, **196**, 335–340.
[30] Rubin, R.A., Haskal, Z.J., O'Brien, C.B., Cope, C. and Brass, C.A. (1995). Transjugular intrahepatic portosystemic shunting: Decreased survival for patients with high APACHE II scores. *American Journal of Gastroenterology*, **90**, 556–563.

Thomas W. Faust M. D.
Michael F. Sorrell M. D.
University of Nebraska Medical Center
Liver Transplant Program

Michael F. Sorrell, M. D.
University of Nebraska Medical Center
Liver Transplant Program
600 S. 42nd Street
Omaha, Nebraska 68198–3280

### Is Hepatectomy Necessary in Dealing with Left Hepatolithiasis with Intrahepatic Duct Stricture?

### ABSTRACT

*Sheen-Chen, S-M., Cheng, Y-F., Chou, F-F.* and *Lee, T-Y. (1995). Ductal dilatation and stenting make routine hepatectomy unnecessary for left hepatolithiasis with intrahepatic biliary stricture. Surgery; 117: 32–36.*

*Background:* **Hepatolithiasis with intrahepatic biliary strictures, more common in Southeast Asia than elsewhere, remains a difficult problem to manage. Hepatic resection has recently been advocated as one of the treatment modalities for hepatolithiasis; however, this procedure is not without risk. This study was designed to achieve complete clearance of the stones, eliminate bile stasis, and avoid the potential risks of hepatic resection in the patient with hepatolithiasis and intrahepatic biliary stricture.**
*Methods:* **In this prospective clinical trial 13 patients with retained left hepatolithiasis and intrahepatic biliary strictures were included. All the patients met the following criteria: (1) initial surgical procedure for hepatolithiasis, (2) normal gross findings of the left liver, and**

